# Optimising intervention implementation in the DCM™ epic trial (dementia care mapping™: to enable person-centred care in care homes)

**DOI:** 10.1186/1745-6215-16-S2-P185

**Published:** 2015-11-16

**Authors:** Liz Graham, Claire Surr, Sharon Jones, Amanda Farrin, Rebecca Walwyn, Robert Cicero

**Affiliations:** 1 University of Leeds, Leeds, UK; 2 Leeds Beckett University, Leeds, UK; 3 University of Bradford, Bradford, UK

## 

The DCM™ EPIC trial requires participating care homes to identify two staff members willing to be trained in the DCM**™** intervention and thereafter implement three DCM ‘mapping cycles’ during the trial. These staff are known as ‘mappers’. This is no small undertaking, and early pilot work highlighted the need to include additional measures to enhance adherence to some elements of intervention uptake and delivery. Various strategies were thus implemented which comprise: a) provision of enhanced information to prospective staff mappers, b) requirement for staff to detail their understanding of DCM™, c) DCM™ expert contact with mappers ahead of randomisation to ensure eligibility and suitability, d) an expert-supported first cycle of mapping, e) review of mapper and expert feedback after the first cycle to ensure areas of concern can be addressed prior to the next cycle, f) CTU contact with mappers to flag the timing of the next mapping cycle, and address any concerns.

To achieve the above, trials unit staff act as a central point of contact, reviewing mapper consent and materials, monitoring each stage of the process and contacting DCM**™** experts and the Chief Investigator to flag issues and problems in a timely manner.

Although enhancing intervention compliance in this way can be seen to be altering the implementation of the intervention and thus reducing its generalizability, it will be discussed that intervention optimisation is necessary to minimise the likelihood of negative results attributable to poor implementation rather than intervention ineffectiveness.

